# A multi-omics study of magnesium sulfate to improve prognosis in sepsis-related encephalopathy: integrating clinical data-driven network pharmacology

**DOI:** 10.3389/fcimb.2025.1607586

**Published:** 2025-06-09

**Authors:** Yingming Kong, Yanghao Tai, Bin Chen, Meng Zhang, Haoyu Ji, Rongke Feng, Liang Shi, Hao Chen

**Affiliations:** ^1^ Third Hospital of Shanxi Medical University, Shanxi Bethune Hospital, Shanxi Academy of Medical Sciences Tongji Shanxi Hospital, Taiyuan, China; ^2^ Basic Medical College, Shanxi Medical University, Taiyuan, China; ^3^ Department of Neurology, Shanxi Bethune Hospital, Shanxi Academy of Medical Science, Tongji Shanxi Hospital, Third Hospital of Shanxi Medical University, Taiyuan, China

**Keywords:** magnesium sulfate, SAE, mortality, MIMIC-IV database, network pharmacology

## Abstract

**Background:**

Sepsis-associated encephalopathy (SAE) constitutes a significant neurological manifestation of sepsis, characterized by high mortality rates and posing a critical threat to patient outcomes. Magnesium sulfate has multiple effects in the nervous system, including neuroprotection, sedation, anticonvulsant activity, enhanced neuroplasticity, anti - inflammation and promotion of nerve repair. It can regulate calcium homeostasis, exert antioxidant effects, and reduce the release of inflammatory factors, thereby alleviating neuronal damage and neurological deficits. This study integrated MIMIC-IV database and network pharmacology to explore magnesium sulfate’s neuroprotective mechanisms and clinical impact on SAE outcomes.

**Methods:**

Retrospective data from 4,650 SAE patients in MIMIC-IV 3.0 were analyzed. Propensity score matching balanced covariates. Cox models and Kaplan-Meier curves evaluated magnesium sulfate’s association with 28-day all-cause mortality (ACM). Network pharmacology identified magnesium sulfate’s core targets and pathways.

**Results:**

4183 patients (89.96%) received magnesium sulfate during ICU, while 467 (10.04%) did not receive. The 28-day ACM in patients with SAE was 11.05%. After propensity score matching participants with and without magnesium sulfate administration had 28-day ACM of 17.29% and 30.42%, respectively (P < 0.001). Magnesium sulfate administration was associated with reduced 28-day ACM. Subgroup analysis revealed this association differed in several stratification. Network pharmacology revealed magnesium sulfate targets TNF, IL6, IL1B and CXCL8, modulating pathways including inflammatory response, immune regulation, and cellular stress.

**Conclusions:**

Magnesium sulfate use correlates with improved SAE survival, particularly those with comorbid chronic obstructive pulmonary disease and acute kidney injury, and using vasoconstrictors, likely through multi-target modulation of inflammatory response and immune regulation. Prospective studies are needed for validation.

## 1 Introduction

Sepsis, a systemic inflammatory response syndrome (SIRS) triggered by infection, represents one of the most critical conditions in intensive care units (ICUs), with persistently high global incidence and mortality rates (O’Brien et al., 2007; Nunnally, 2016). Sepsis-associated encephalopathy (SAE), a common neurological complication of sepsis, manifests as consciousness impairment, cognitive decline, and electroencephalographic abnormalities (Gofton and Young, 2012; Tauber et al., 2021; Sonneville et al., 2023). This condition affects up to 70% of critically ill septic patients and is independently associated with increased mortality and long-term neurological deficits (Ziaja, 2013; Chung et al., 2020). Despite significant advancements in sepsis diagnosis and treatment, the lack of complete understanding regarding SAE pathogenesis has limited the development of effective clinical interventions.

Magnesium sulfate, a widely used magnesium salt, has been extensively employed in diverse clinical settings, including preeclampsia management, arrhythmia correction, and adjunctive treatment for neurological disorders (Cohen and Kitzes, 1983; Duley et al., 2010; Li et al., 2017). Recent studies have increasingly focused on its potential therapeutic value in sepsis management, particularly its neuroprotective effects against SAE (Esen et al., 2005; Li et al., 2019). As an essential mineral, magnesium participates in multiple physiological processes such as nerve conduction, inflammatory regulation, and cellular energy metabolism (Cazzola et al., 2024; Kumar et al., 2024). However, systematic investigations into magnesium sulfate’s specific mechanisms and molecular targets for improving SAE prognosis remain insufficient.

Previous research on sepsis-associated encephalopathy (SAE) has predominantly focused on single biomarkers or pathway-specific analyses, often overlooking the complex multisystem interactions and polygenic nature of SAE pathophysiology. Most existing studies are constrained by small sample sizes or a lack of mechanistic insights linking therapeutic interventions to clinical outcomes in SAE patients. Furthermore, traditional methodologies remain inadequate in delineating the neuroprotective effects of potential treatments such as magnesium sulfate. This study addresses these limitations by adopting a multi-omics framework integrated with network pharmacology.

The MIMIC-IV database, a publicly accessible repository containing comprehensive ICU patient data, provides robust resources for critical care research (Johnson et al., 2023). Diverging from conventional approaches, our analysis leverages the large-scale patient cohorts in the MIMIC-IV database to systematically evaluate magnesium sulfate’s impact on SAE prognosis. By employing network pharmacology, we systematically constructed SAE-specific gene regulatory networks and protein-protein interaction maps, identifying hub genes and key pathways modulated by magnesium sulfate. This represents a paradigm shift from investigating isolated drug effects on disease outcomes to deciphering the systemic influence of magnesium sulfate on the interconnected SAE network. The innovative application of multi-omics integration in SAE research not only provides a robust tool for uncovering potential therapeutic targets and clinically biomarkers but also establishes a methodological framework for precision medicine in sepsis-related neurological complications.

## 2 Methods

### 2.1 Clinical study design

Employing data drawn from the MIMIC-IV database (version 3.0), the study adopts a retrospective observational cohort analysis approach to evaluate the research question. This publicly available database contains 94,458 ICU admission records. The author, B.C., who holds a certification from the Collaborative Institutional Training Initiative (CITI), collected the data for this study. Given that anonymized data were used for this secondary analysis, informed consent was not sought. The dataset was accessible to researchers who met the criteria for data utilization. Adhering to the ethical standards set forth by the Helsinki Declaration, this study also complied with the STROBE guidelines, which are designed to enhance the quality of reporting for observational research in the field of epidemiology.

**Figure 1:**
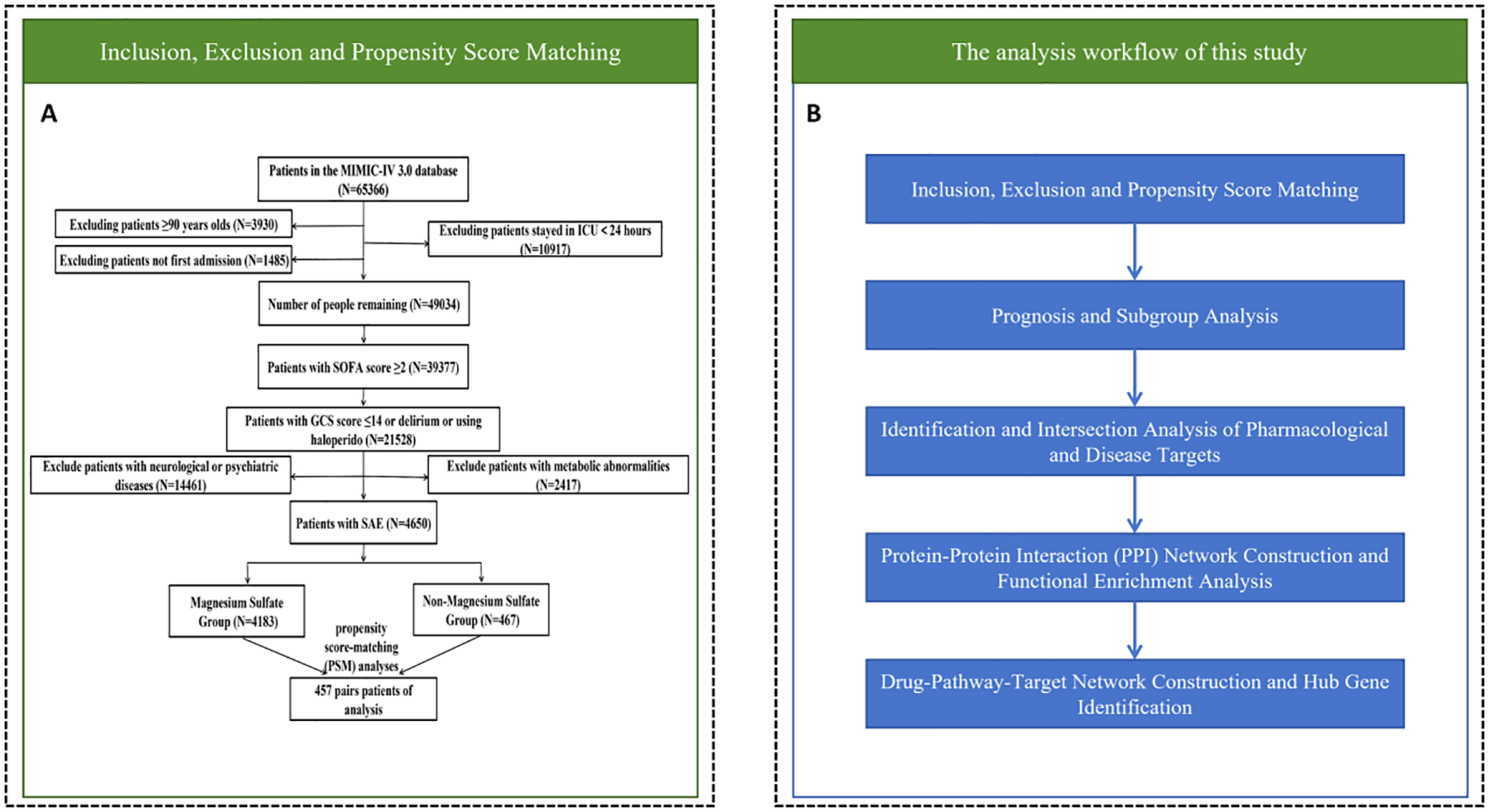
Research flow chart. **(A)** Inclusion exclusion and propensity score matching. **(B)** Prognostic and network pharmacologic analysis.

From the MIMIC-IV database, the research encompassed 65366 individuals over 18 years old hospitalized in the ICU. The exclusion criteria for our study were established as: (1) Patients younger than 18 or older than 90 (n = 3930); (2) ICU stays shorter than 24 hours (n = 10917); (3) non-primarily admitted patients and those not admitted for the first time to the ICU (n = 1485); (4) patients with SOFA score< 2 (n = 9657); (5) patients with GCS score < 15 or without a diagnosis of delirium (ICD codes 2930, 2931, F05) or without a treatment with haloperidol (n = 17849); (6) patients diagnosed with other psychiatric and neurological disorders (cerebral hemorrhage, ischemic stroke, epilepsy, intracranial infections, traumatic brain injury, and other cerebrovascular disorders, metabolic encephalopathy, hepatic encephalopathy, hypertensive encephalopathy, chronic alcohol or drug abuse, and psychiatric and other neurological disorders) (n = 14461). (7) patients diagnosed with hyponatremia (< 120 mmoL/L), hyperglycemia (> 180 mg/dL), hypoglycemia (< 54 mg/dL), and PaCO_2_ ≥ 80 mmHg (n = 2417). The final sample consisted of 4650 patients (**Figure 1A**).

#### 2.1.1 Data extraction

Patient data, encompassing both clinical and demographic aspects, were retrieved through the execution of SQL queries utilizing the PostgreSQL database management system (version 13.7.2), complemented by the Navicat Premium client tool (version 16). The initial assessment captured a range of attributes such as age, gender, weight, alongside the mean values of critical physiological parameters, including respiratory rate, heart rate, pulse oximetry saturation of oxygen (SpO2), and mean arterial blood pressure all recorded for the first time in ICU admission. Additionally, a comprehensive set of laboratory indicators was examined, consisting of white blood cell (WBC) counts, serum chloride, sodium, potassium levels, total calcium levels, prothrombin time (PT), international normalized ratio (INR), platelet (PLT) counts, hemoglobin, serum glucose, anion gap creatinine and serum urea nitrogen (BUN). Therapeutic measures include invasive mechanical ventilation (IMV), continuous renal replacement therapy (CRRT), vasopressor, dexmedetomidine, propofol and hydrocortisone. The assessment tools utilized in this study comprised Sequential Organ Failure Assessment (SOFA), Charlson Comorbidity Index (CCI) and the Acute Physiology Score III (APS III). Comorbid features included hypertension, diabetes, myocardial infarction, heart failure, COPD, hyperlipidemia, acute renal failure, respiratory failure, and peripheral vascular disease.

#### 2.1.2 Outcomes

The study’s primary outcomes were the rates of mortality from all causes mortality (ACM) at 28 days.

#### 2.1.3 Statistical analysis

Continuous variables following a normal distribution were presented using means ± standard deviations. For continuous variables that were not normally distributed, medians and interquartile ranges were used. Categorical data were described using frequency counts and percentages. Non-parametric data were assessed via the Mann-Whitney U test. Parametric data were analyzed using the student’s t-test. Participants were stratified into two cohorts according to their exposure to magnesium sulfate. Assessment of the estimated mortality rates and related disparities was conducted using Kaplan-Meier survival analysis. Subsequently, the statistical significance across groups was evaluated by the Log-Rank test. Propensity Score Matching (PSM) was utilized as a methodological approach to mitigate the impact of potential confounding factors. Utilizing demographic and clinical characteristics, we calculated propensity scores through a logistic regression model. The propensity score matching process was conducted as follows: A random seed of 123 was set to ensure replicability. Propensity scores were estimated using all covariates. Then, 1:1 nearest neighbor matching without replacement was performed, with a caliper width set at 0.25 times the standard deviation of the propensity score. This process yielded a matched cohort for subsequent analysis. In the propensity score matching process, we meticulously balanced the covariates by selecting a comprehensive set of matching variables. These variables included demographic characteristics ((e.g., gender, age, weight), vital signs, laboratory indicators (e.g., blood test results), the presence of comorbidities, therapeutic measures (e.g., types of treatments administered), and various scores (e.g., severity scores).This careful selection of matching variables aimed to minimize bias and ensure comparability between groups, thereby strengthening the validity of the study findings. To assess covariate balance before and after matching, we computed the absolute standardized mean difference (SMD). Cox proportional hazards regression was then applied to examine the association between magnesium sulfate treatment and the key outcomes of interest. For the multivariate Cox models, a selection of covariates was made based on established literature and their clinical significance. These included: Model 1 which was unadjusted, Model 2, adjusted for weight, gender, age, and a comprehensive model (Model 3) that further controlled for age, gender, weight, heart rate, CRRT, mechanical ventilation, SOFA, APSIII, CCI, mean arterial pressure, respiratory rate, SpO2, BUN,WBC, PT, PLT, Hb, INR, sodium, potassium, total calcium, chloride, glucose, anion gap, creatinine, vasopressor, dexmedetomidine, propofol, hydrocortisone, hypertension, diabetes, myocardial infarction, heart failure, COPD, hyperlipidemia, acute renal failure, respiratory failure, and peripheral vascular disease. Based on various patient characteristics, subgroup analyses were conducted to elucidate the impact of magnesium sulfate use, such as age, CRRT, mechanical ventilation, gender, SOFA, vasopressor, dexmedetomidine, propofol, hydrocortisone, hypertension, diabetes, myocardial infarction, heart failure, COPD, hyperlipidemia, acute renal failure, respiratory failure, and peripheral vascular disease. Statistical analyses were accomplished utilizing SPSS 25.0 software (developed by IBM, based in the USA) alongside R 4.1.2 (provided by the R Foundation). Statistical significance was determined using a p-value threshold set at less than 0.05.

### 2.2 Network pharmacology

#### 2.2.1 Identification and intersection analysis of pharmacological and disease targets

Therapeutic targets of magnesium sulfate were systematically compiled from the GeneCards database (https://www.genecards.org/). The canonical SMILES notation (CID 24083) was retrieved from PubChem (https://pubchem.ncbi.nlm.nih.gov/) for subsequent structure-based target prediction. SAE-associated targets were identified through comprehensive mining of the GeneCards database and analysis of the GSE167610 dataset from Gene Expression Omnibus (GEO, https://www.ncbi.nlm.nih.gov/geo/). Differential expression analysis between SAE model and control groups in Mus musculus was performed using GEO2R with stringent criteria (adjusted p-value <0.05 and |log2(fold change)| >1). A Venn diagrammatic approach was employed to identify putative therapeutic targets through intersection analysis of magnesium sulfate-targeted proteins and SAE-associated genes using the Venny 2.1 platform (https://bioinfogp.cnb.csic.es/tools/venny/) (**Supplementary Figures 1A, B**).

#### 2.2.2 Protein-protein interaction network construction and functional enrichment analysis

The STRING database (https://string-db.org/, version 11.5) was utilized to construct a high-confidence PPI network (interaction score ≥0.7) for intersected targets, with parameter settings restricted to Homo sapiens to ensure clinical relevance. Multi-omics enrichment analysis was conducted through: Gene Ontology (GO) annotation covering biological processes, molecular functions, and cellular components. Kyoto Encyclopedia of Genes and Genomes (KEGG) pathway mapping (**Supplementary Figures 1C, D**).

#### 2.2.3 Drug-pathway-target network construction and hub gene identification

An integrated drug-pathway-target network was visualized using Cytoscape (v3.10.3). Topological analysis employing the CytoHubba plugin identified hub genes through Maximal Clique Centrality (MCC) algorithm, with the top 10 nodes selected as core targets (**Supplementary Figures 1E, F**).

In summary, the overall workflow of this study began with patient inclusion, exclusion criteria application, and propensity score matching, as depicted in **Figure 1A**. Subsequently, the study progressed through prognostic analysis and subgroup analyses, then advanced to the identification and intersection analysis of pharmacological and disease targets. Following this, a protein-protein interaction (PPI) network was constructed, and functional enrichment analysis was performed. Finally, the workflow culminated in the construction of a drug-pathway-target network and the identification of hub genes (**Figure 1B**).

## 3 Results

### 3.1 Baseline characteristics

Based on the exclusion criteria, 4650 eligible ICU individuals were included in this research. Among them, 4183 patients (89.96%) were on magnesium sulfate after ICU admission and 467 (10.04%) were not on magnesium sulfate during this period. Between the two groups, significant differences were found in terms of APSIII score, Charlson comorbidity index, gender, mean arterial blood pressure, SpO_2_, PLT, potassium, chloride, anion gap, free calcium, PT, INR, BUN, creatinine, CRRT, hypertension, diabetes, myocardial infarction, heart failure, hyperlipidemia, acute renal failure, and respiratory failure, IMV, vasopressor, dexmedetomidine and propofol (p < 0.05). The 28-day ACM for individuals with SAE was 11.05%. After adjustment using PSM, 457 patients on magnesium sulfate and 457 patients not on magnesium sulfate were included. The characteristics and results of the original and PSM cohorts are shown in **Table 1**. Within the propensity-matched cohort, the standardized mean differences of all baseline covariates were maintained below 0.10, thereby confirming negligible imbalance between the groups. The 28-day mortality rates after PSM were 17.29% and 30.42% for magnesium sulfate-using and non-magnesium sulfate-using participants, respectively (P < 0.001).

**Table 1:** Baseline characteristics.

Variable	Before PSM				After PSM			
	Total (n = 4650)	Non-users (n = 467)	Users (n = 4183)	*P*	Total (n = 914)	Non-users (n = 457)	Users (n = 457)	*P*
Gender, Male, n (%)	3021 (64.97)	268 (57.39)	2753 (65.81)	<0.001	521 (57.00)	263 (57.55)	258 (56.46)	0.738
Age, Mean ± SD	67.83 ± 14.07	67.19 ± 15.73	67.90 ± 13.88	0.347	67.29 ± 15.74	67.08 ± 15.80	67.50 ± 15.68	0.690
Weight, Mean ± SD	85.18 ± 22.19	83.18 ± 24.53	85.41 ± 21.91	0.061	82.35 ± 24.90	83.29 ± 24.67	81.42 ± 25.11	0.255
Vital Signs								
Heart Rate, Mean ± SD	84.85 ± 14.11	86.02 ± 17.16	84.71 ± 13.72	0.111	86.14 ± 16.89	86.22 ± 17.19	86.06 ± 16.59	0.884
Mean arterial pressure, Mean ± SD	74.32 ± 9.73	75.71 ± 12.03	74.16 ± 9.43	0.007	75.01 ± 11.55	75.65 ± 12.11	74.37 ± 10.93	0.095
Respiratory rate, Mean ± SD	19.73 ± 6.65	19.75 ± 6.53	19.29 ± 6.44	0.314	19.80 ± 5.74	19.95 ± 4.85	19.65 ± 6.51	0.429
SpO_2_, Mean ± SD	97.44 ± 7.35	96.43 ± 2.23	97.55 ± 7.71	0.002	97.30 ± 16.24	96.44 ± 2.23	98.15 ± 22.84	0.113
Laboratory indicators								
WBC, Mean ± SD	13.28 ± 7.85	13.55 ± 9.75	13.25 ± 7.61	0.523	13.35 ± 9.38	13.44 ± 9.62	13.27 ± 9.13	0.782
PLT, Mean ± SD	181.13 ± 90.35	209.40 ± 122.50	177.98 ± 85.46	<0.001	205.73 ± 113.60	208.15 ± 122.20	203.32 ± 104.37	0.521
Hemoglobin, Mean ± SD	10.35 ± 1.70	10.20 ± 1.97	10.36 ± 1.67	0.076	10.25 ± 1.96	10.25 ± 1.96	10.26 ± 1.96	0.911
Sodium, Mean ± SD	138.17 ± 3.63	138.15 ± 4.71	138.17 ± 3.49	0.931	137.98 ± 4.75	138.10 ± 4.63	137.86 ± 4.87	0.439
Potassium, Mean ± SD	4.32 ± 0.51	4.40 ± 0.66	4.31 ± 0.49	0.009	4.36 ± 0.64	4.39 ± 0.66	4.34 ± 0.61	0.233
Calcium, Mean ± SD	8.24 ± 0.62	8.39 ± 0.75	8.23 ± 0.60	<0.001	8.34 ± 0.79	8.39 ± 0.75	8.30 ± 0.83	0.119
Chloride, Mean ± SD	105.50 ± 5.00	103.07 ± 6.25	105.77 ± 4.77	<0.001	103.41 ± 6.27	103.05 ± 6.17	103.77 ± 6.34	0.081
Glucose, Mean ± SD	123.98 ± 24.66	125.63 ± 26.67	123.80 ± 24.42	0.157	125.71 ± 26.63	125.88 ± 26.52	125.55 ± 26.78	0.853
Anion gap, Mean ± SD	12.95 ± 3.58	15.31 ± 4.11	12.68 ± 3.42	<0.001	14.96 ± 4.16	15.21 ± 4.04	14.71 ± 4.27	0.069
PT, Mean ± SD	15.60 ± 5.66	17.45 ± 9.60	15.39 ± 4.99	<0.001	17.09 ± 8.54	17.30 ± 9.19	16.88 ± 7.85	0.459
INR, Mean ± SD	1.43 ± 0.56	1.62 ± 1.01	1.40 ± 0.47	<0.001	1.58 ± 0.89	1.60 ± 0.97	1.56 ± 0.80	0.507
BUN, Mean ± SD	24.17 ± 19.86	38.26 ± 29.87	22.60 ± 17.73	<0.001	35.28 ± 28.46	36.96 ± 28.36	33.61 ± 28.50	0.075
Creatinine, Mean ± SD	1.36 ± 1.43	2.19 ± 2.58	1.26 ± 1.21	<0.001	1.98 ± 2.28	2.12 ± 2.50	1.84 ± 2.03	0.067
Comorbidity								
Hypertension, n (%)	2212 (47.57)	156 (33.40)	2056 (49.15)	<0.001	319 (34.9)	154 (33.70)	165 (36.11)	0.445
Diabetes Mellitus, n (%)	1317 (28.32)	111 (23.77)	1206 (28.83)	0.021	222 (24.29)	108 (23.63)	114 (24.95)	0.644
Heart Failure, n (%)	1171 (25.18)	150 (32.12)	1021 (24.41)	<0.001	272 (29.76)	146 (31.95)	126 (27.57)	0.148
Myocardial infarction, n (%)	374 (8.04)	25 (5.35)	349 (8.34)	0.024	45 (4.92)	23 (5.03)	22 (4.81)	0.878
COPD, n (%)	267 (5.74)	27 (5.78)	240 (5.74)	0.969	62 (6.78)	27 (5.91)	35 (7.66)	0.293
Hyperlipidemia, n (%)	2296 (49.38)	158 (33.83)	2138 (51.11)	<0.001	334 (36.54)	156 (34.14)	178 (38.95)	0.131
Acute renal failure, n (%)	1315 (28.28)	209 (44.75)	1106 (26.44)	<0.001	397 (43.44)	203 (44.42)	194 (42.45)	0.548
Respiratory failure, n (%)	983 (21.14)	158 (33.83)	825 (19.72)	<0.001	289 (31.62)	153 (33.48)	136 (29.76)	0.227
Peripheral vascular disease, n (%)	200 (4.3)	21 (4.50)	179 (4.28)	0.826	41 (4.49)	20 (4.38)	21 (4.60)	0.873
Therapeutic measures								
CRRT, n (%)	194 (4.17)	28 (6.00)	166 (3.97)	0.038	59 (6.46)	28 (6.13)	31 (6.78)	0.686
IMV, n (%)	2829 (60.84)	160 (34.26)	2669 (63.81)	<0.001	328 (35.89)	158 (34.57)	170 (37.20)	0.408
Dexmedetomidine, n (%)	937 (20.15)	36 (7.71)	901 (21.54)	<0.001	86 (9.41)	36 (7.88)	50 (10.94)	0.113
Propofol, n (%)	3073 (66.09)	98 (20.99)	2975 (71.12)	<0.001	220 (24.07)	97 (21.23)	123 (26.91)	0.044
Vasopressor, n (%)	3481 (74.86)	162 (34.69)	3319 (79.34)	<0.001	348 (38.07)	162 (35.45)	186 (40.70)	0.102
Hydrocortisone, n (%)	273 (5.87)	29 (6.21)	244 (5.83)	0.743	55 (6.02)	28 (6.13)	27 (5.91)	0.889
Scores								
SOFA, Mean ± SD	6.26 ± 3.08	6.33 ± 3.68	6.25 ± 3.01	0.655	6.36 ± 3.61	6.31 ± 3.70	6.42 ± 3.51	0.660
APS III, Mean ± SD	49.35 ± 23.43	55.35 ± 25.07	48.68 ± 23.15	<0.001	55.10 ± 24.59	55.14 ± 25.16	55.06 ± 24.04	0.959
CCI, Mean ± SD	4.81 ± 2.80	5.87 ± 3.30	4.70 ± 2.71	<0.001	5.74 ± 3.18	5.85 ± 3.32	5.62 ± 3.04	0.289
28 days ACM, n (%)	4650 (11.05)	467 (30.41)	4183 (8.89)	<0.001	914 (23.85)	457 (30.42)	457 (17.29)	<0.001

### 3.2 Survival correlation

**Figure 2:**
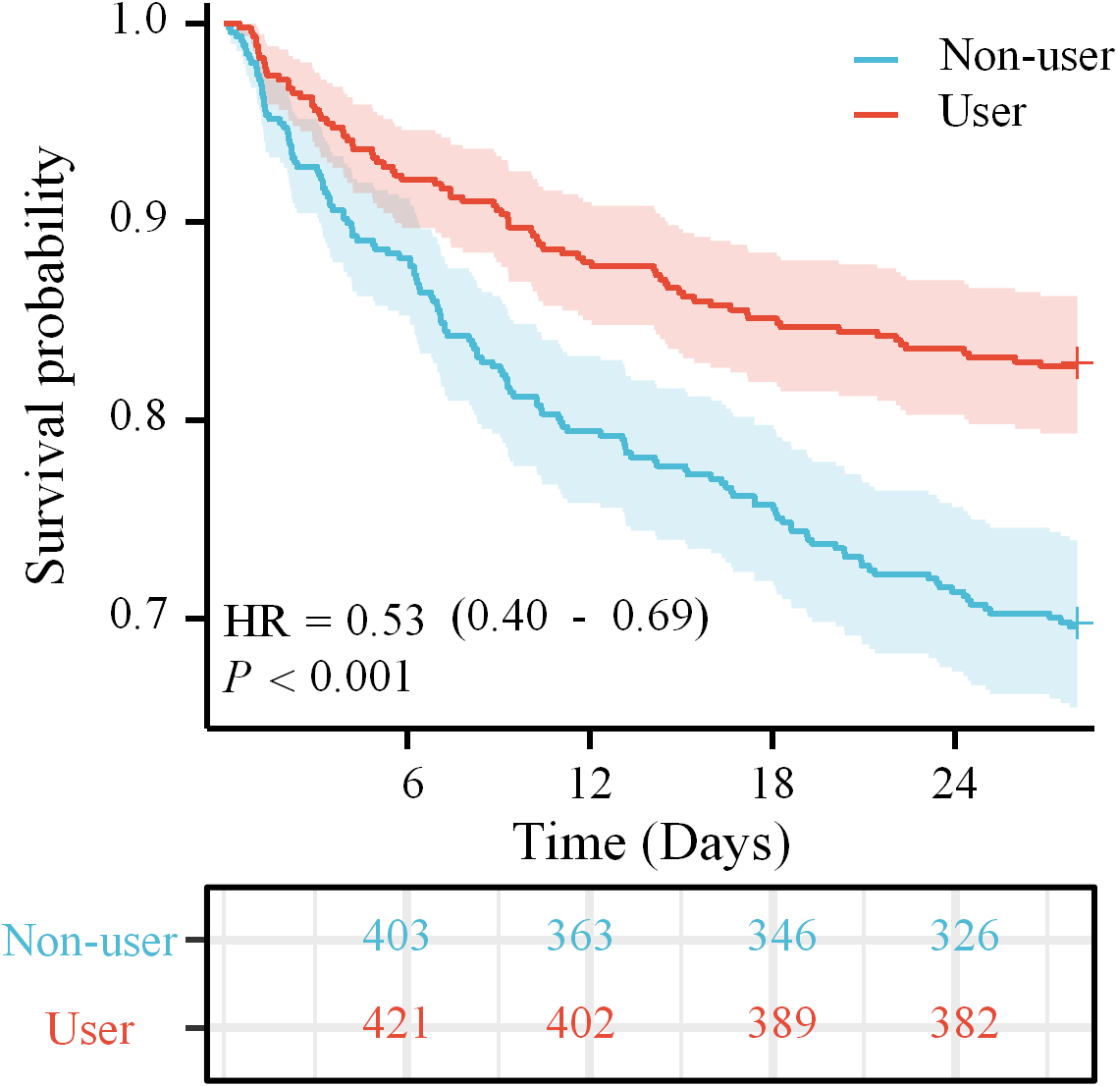
KM curve analysis of magnesium sulfate use in relation to 28-day survival in SAE patients.

Both the Kaplan-Meier curvet and the log-rank test revealed compared to non-users, magnesium sulfate-users had higher probability of survival in **Figure 2**. Multivariate analysis indicated the utilization of magnesium sulfate was correlated to lower 28-day ACM (HR = 0.57, 95% CI 0.42-0.76, P < 0.001) in individuals with SAE (**Table 2**). To further elucidate the overall survival differences between magnesium sulfate users and non-users, for differences in survival rates at specific time points (6d, 12d, 18d, 24d), there was overall significance between the two groups(**Supplementary Figure 2**).

**Table 2:** Univariate and multivariate Cox regression modeling elucidating the association between magnesium sulfate and all-cause mortality in patients with SAE.

Outcome	HR (95% CI)					
	Model 1	P Value	Model 2	P Value	Model 3	P Value
28 days ACM	0.53 (0.40-0.69)	<0.001	0.54 (0.40-0.71)	<0.001	0.57 (0.42-0.76)	<0.001

Model 1 was adjusted for none.

Model 2 was adjusted for age, gender, weight.

Model 3 was adjusted for age, gender, weight, CRRT, hypertension, diabetes, heart failure, myocardial infarction, COPD, hyperlipidemia, acute renal failure, respiratory failure, peripheral vascular disease, mechanical ventilation, SOFA, APSIII, CCI, heart rate, mean arterial pressure, respiratory rate, SpO2, white blood cell, platelet count, hemoglobin, sodium, potassium, total calcium, chloride, bloodglucose, anion gap, prothrombin time, international normalized ratio, BUN, creatinine, dexmedetomidine, propofol, vasopressor, hydrocortisone.

### 3.3 Subgroup analysis

**Figure 3:**
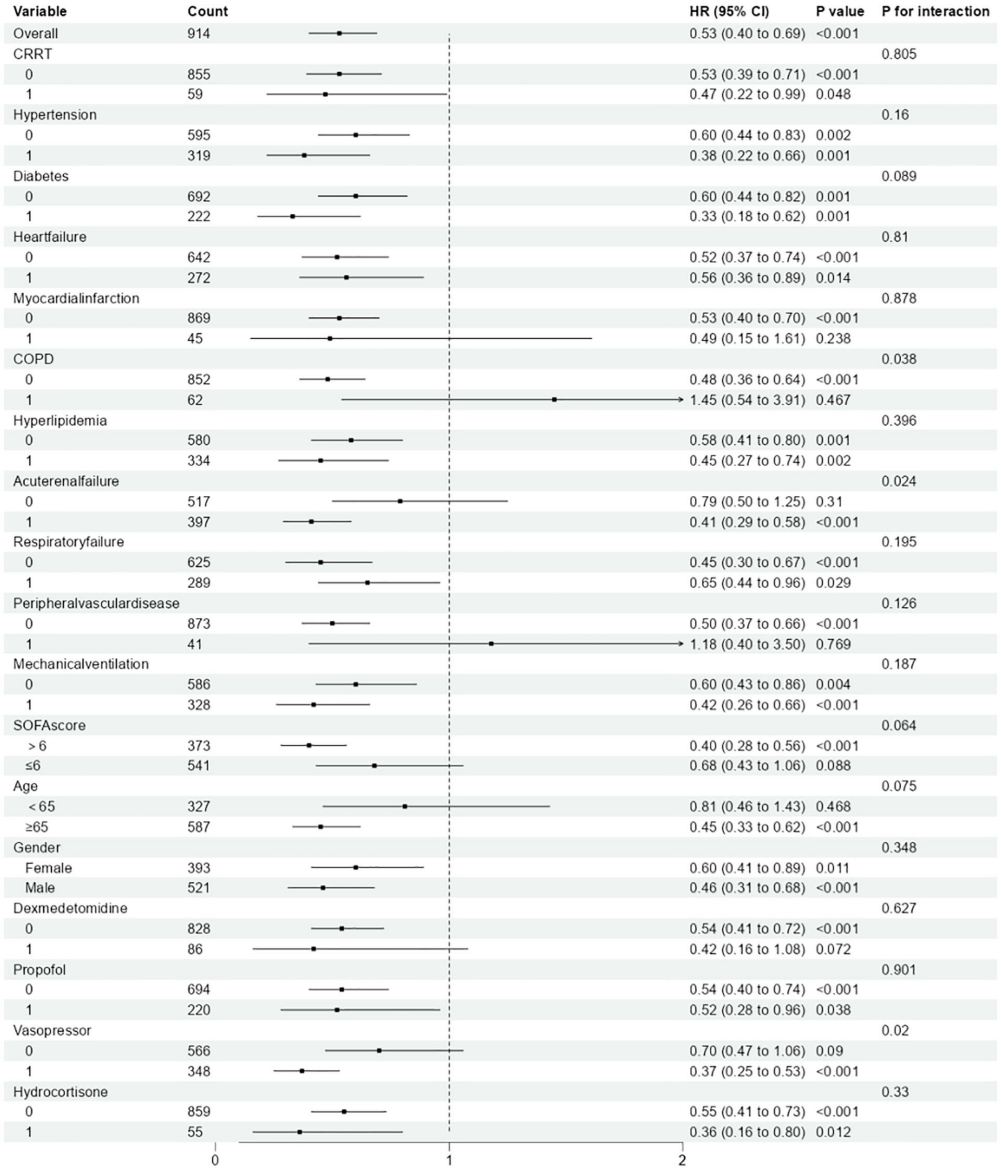
Association between magnesium sulfate use and all-cause mortality in patients with SAE between different subgroups.(The “P value” represents the statistical significance of the association between magnesium sulfate use and all-cause mortality within each subgroup. The “P for interaction” assesses whether the effect of magnesium sulfate varies significantly across different subgroups.).

Stratified analyses were implemented to estimate the relationship between magnesium sulfate treatment and ACM among individuals with SAE across various subgroups (**Figure 3**). The analyses were stratified by age, CRRT, mechanical ventilation, gender, SOFA, vasopressor, dexmedetomidine, propofol, hydrocortisone, hypertension, diabetes, myocardial infarction, heart failure, COPD, hyperlipidemia, acute renal failure, respiratory failure, and peripheral vascular disease. Research findings indicate that, based on the current dataset, there is insufficient evidence to reject the null hypothesis of no interaction between magnesium sulfate treatment and most subgroups (P for interaction>0.05). In our subgroup analysis, we observed variations in the effect size of magnesium sulfate on 28-day all-cause mortality (ACM) across different subgroups. The hazard ratio (HR) for magnesium sulfate use ranged from 0.33 to 1.45, indicating a diverse impact depending on specific patient characteristics. Notably, significant interactions were found in patients with chronic obstructive pulmonary disease (COPD) (P for interaction = 0.038), acute renal failure (ARF) (P for interaction = 0.024), and those using vasopressors (P for interaction = 0.02). In these subgroups, the survival benefit associated with magnesium sulfate was more pronounced. In contrast, other subgroups such as those with hypertension (P for interaction = 0.16), diabetes (P for interaction = 0.089), and heart failure (P for interaction = 0.81) did not show statistically significant interactions. These findings suggest that magnesium sulfate may have a more substantial impact on survival in patients with specific comorbidities or those requiring certain therapeutic measures. In addition, we performed Kaplan-Meier analyses of differences in survival in relation to specific patient characteristics. The results showed that among patients on magnesium sulfate, those with comorbid COPD, ARF, and use of vasopressors had a worse prognosis (**Supplementary Figure 3**).

### 3.4 Drug-disease intersection target screening

**Figure 4:**
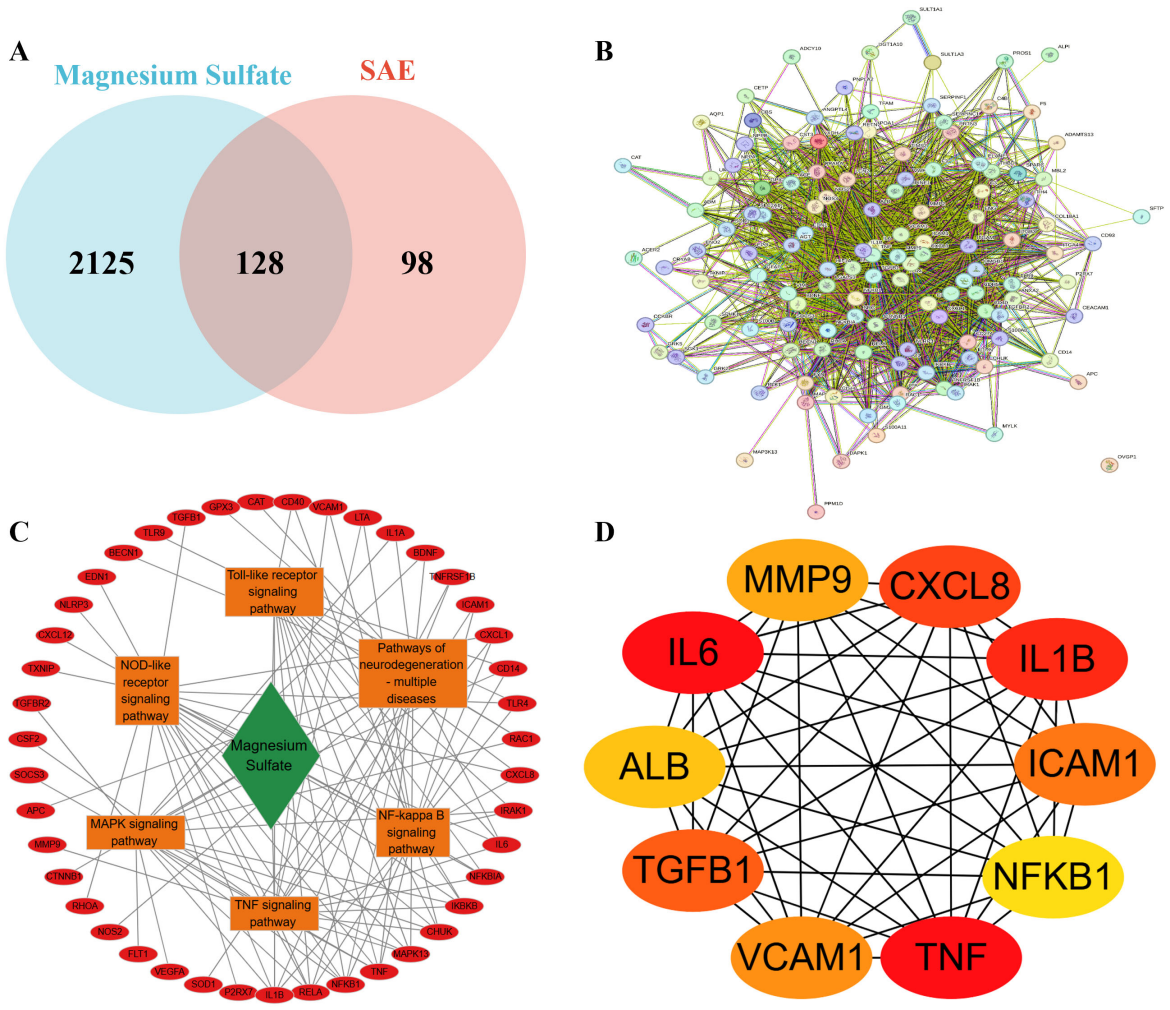
Screening of magnesium sulfate to improve SAE targets **(A)** Intersection of Isoproterenol Therapeutic Targets and SAE Disease Targets; **(B)** Construction of 128 cross-protein interaction networks, nodes represent proteins. Connections represent protein-protein binding; **(C)** The “drug-target-pathway” network is constructed with a red circle for the target, a green diamond for the drug, and an orange box for the pathway; **(D)** Core targets were screened and colors indicate ordering.

A total of 2253 potential target of magnesium sulfate were obtained from the GeneCards database. Meanwhile, 157 target genes related to SAE were screened from the GeneCards database. 86 DEGs were identified from GSE167610 dataset. After standardization by the UniProt database, 226 disease-related targets were determined. Venny analysis yielded 128 drug-disease intersection targets (**Figure 4A**), suggesting that these targets may mediate the therapeutic effect of magnesium sulfate on SAE. The gene set details are displayed in **Supplementary Table Sheet 1**.

### 3.5 Protein-protein interaction network analysis

The PPI network constructed based on the STRING database (**Figure 4B**) showed that the 128 candidate targets formed a highly associated interaction network (network density = 31.2, number of edges = 1980), whose topological parameters were significantly better than those of the random network (expected number of edges = 581, p < 1.0e - 16). The network clustering coefficient reached 0.799, indicating significant synergistic mechanisms among the targets. The top 10 core targets identified by MCC algorithm in the CytoHubba plugin were TNF, IL6, IL1B, CXCL8, TGFB1, ICAM1, VCAM1, MMP9, ALB and NFKB1 (**Figures 4C, DC**). **Supplementary Table Sheet 3** describes in detail the pathway and drug target interactions mentioned in **Figure 4C**. A complete list of abbreviations and functions for the 10 core targets is provided in **Supplementary Table Sheet 4**. The expression of some of the core targets is shown in **Supplementary Table Sheet 2**. Notably, the first seven targets were all pro-inflammatory cytokines and key molecules in their signal transduction, suggesting that neuroinflammation regulation may be the core mechanism of magnesium sulfate’s neuroprotective effect.

### 3.6 Gene function analysis

**Figure 5:**
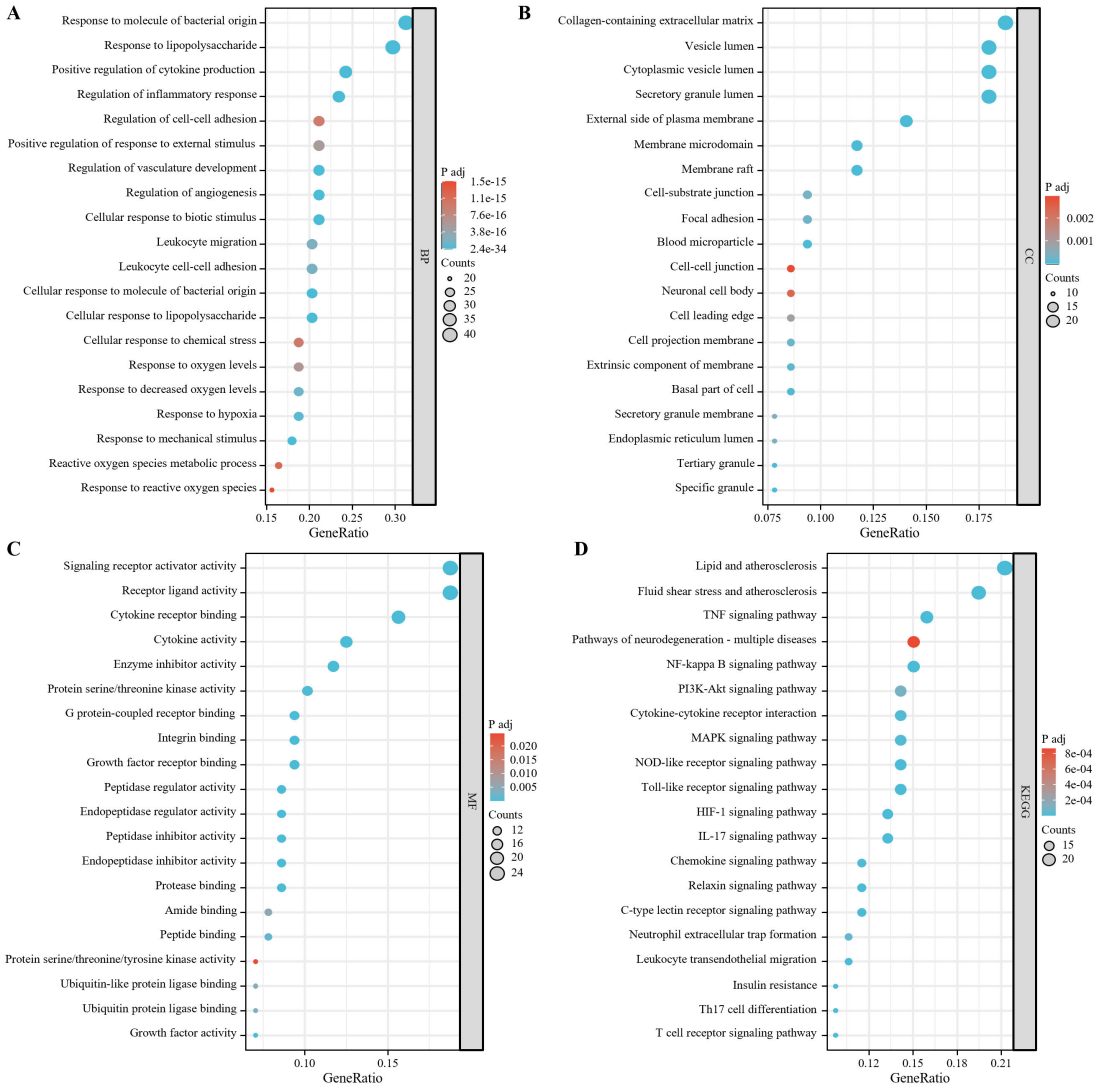
128 target enrichment analysis **(A)** biological process; **(B)** cellular component; **(C)** molecular function;**(D)** KEGG.

The biological process analysis showed that the 128 targets were significantly enriched in processes such as response to molecule of bacterial origin, response to lipopolysaccharide, positive regulation of cytokine production and regulation of inflammatory response (**Figure 5A**). The cellular component analysis indicated that the target proteins were mainly located in collagen-containing extracellular matrix, vesicle lumen, cytoplasmic vesicle lumen, secretory granule lumen (**Figure 5B**). At the molecular function level, the targets were significantly enriched in functional modules such as signaling receptor activator activity, receptor ligand activity, cytokine receptor binding and cytokine activity (**Figure 5C**). The pathway analysis showed that the candidate targets were primarily associated with the subsequent key signaling pathways (**Figure 5D**): inflammatory response, immune regulation, and cellular stress, including NF-κB, MAPK, PI3K-Akt, Toll-like receptor, and NOD-like receptor signaling pathways. Together, these pathways are involved in pro-inflammatory factor release (TNF, IL-17), immune cell activation (Th17 differentiation, T-cell receptor signaling), and regulation of endothelial barrier function (leukocyte migration across the endothelium).

## 4 Discussion

In our study, the 28-day all-cause mortality (ACM) among SAE patients was 11.05%. After propensity score matching, the 28-day ACM for SAE individuals increased to 23.85%. We investigated the association between magnesium sulfate initiation and mortality in SAE patients. Multivariable-adjusted Cox regression analysis revealed that magnesium sulfate use was associated with reduced all-cause mortality in this cohort. Subgroup analyses stratified by sex, use of continuous renal replacement therapy (CRRT), mechanical ventilation, dexamethasone, dexmedetomidine, propofol, hydrocortisone, and comorbidities (hypertension, diabetes, heart failure, myocardial infarction, chronic obstructive pulmonary disease [COPD], hyperlipidemia, respiratory failure, and peripheral vascular disease) demonstrated consistent results. Significant interactions were observed in subgroups stratified by vasopressor use, acute kidney injury (AKI), and COPD. This interaction may be attributed to sepsis-induced hypotension, which exacerbates cerebral hypoperfusion. Vasopressors improve blood pressure and cerebral perfusion, potentially enhancing magnesium sulfate’s neuroprotective effects by stabilizing hemodynamics (Cheung et al., 2025). AKI patients frequently exhibit magnesium dysregulation, with hypomagnesemia impairing neuronal conduction and mitochondrial function (Li et al., 2024). Magnesium supplementation corrects hypomagnesemia, restoring neuronal excitability and cellular homeostasis (Shindo et al., 2015; Shen et al., 2019). Patients without COPD may see a greater reduction in SAE mortality from magnesium sulfate than those with COPD. This could be due to the chronic inflammation, electrolyte disturbances, and impaired respiratory function often found in COPD patients, which may affect the pharmacokinetics and pharmacodynamics of magnesium sulfate (Stroda et al., 2018). Additionally, magnesium sulfate might be more effective in non - COPD patients through bronchodilation or inflammation regulation. In contrast, long - term airway remodeling in COPD patients could reduce its efficacy or even cause the opposite effect. However, the COPD group’s small sample size necessitates further validation with a larger sample. These findings can help clinicians consider whether to use magnesium sulfate as adjuvant therapy based on the patient’s specific comorbidities (such as COPD or ARF) or treatment needs (such as the use of vasopressors). This helps to achieve more personalized treatment plans. Moreover, given limited resources, prioritizing the use of magnesium sulfate in these specific subgroups may be more effective in reducing mortality rates and improving patient outcomes. In addition, these results emphasize the importance of further research in these specific subgroups to validate the efficacy and safety of magnesium sulfate and optimize its clinical application.

Compared to other neuroprotective agents under investigation for SAE, such as NMDA receptor antagonists (e.g., ketamine) or cytokine-specific biologics (e.g., anti-IL-6 antibodies), magnesium sulfate offers a multimodal mechanism of action (Xu et al., 2025). While ketamine primarily targets excitotoxicity and biologics suppress specific cytokines, magnesium sulfate concurrently mitigates neuroinflammation (via NF-κB inhibition), stabilizes BBB integrity (through MMP9 downregulation), and corrects metabolic disturbances (e.g., hypomagnesemia) (Wang et al., 2025). This polypharmacological profile may explain its survival benefit in our cohort, aligning with emerging evidence that combinatorial therapies outperform single-pathway interventions in sepsis-related organ dysfunction. Sepsis-associated encephalopathy (SAE) pathogenesis involves excessive release of pro-inflammatory cytokines (e.g., TNF-α, IL-1β, IL-6), which breach the blood-brain barrier (BBB), activate microglia and astrocytes, and drive neuroinflammation (Consales and De Gaudio, 2005). Magnesium exerts neuroprotection through multiple mechanisms. As a voltage-dependent NMDA receptor antagonist, it mitigates excitotoxicity and promotes synaptic plasticity (Fekete and Wang, 2025). It suppresses inflammatory mediator production and neuroinflammation (Patel et al., 2024). It stabilizes neuronal ion homeostasis, regulates neurotransmitter release, preserves BBB integrity, and exerts antioxidant effects via modulation of intracellular signaling pathways (Saver et al., 2015; Maier et al., 2022; Scibior et al., 2024).

Network pharmacology identified the top 10 targets—TNF, IL6, IL1B, CXCL8, TGFB1, ICAM1, VCAM1, MMP9, ALB, and NFKB1—highlighting pathways linked to neuroinflammation, oxidative stress, and BBB dysfunction. In addition, the enrichment of neurodegenerative disease-related pathways and insulin resistance suggests that SAE may involve an interaction between metabolic imbalance and neuroinflammation. The above results suggest that magnesium sulfate may intervene in the pathological process of SAE by synergistically modulating the inflammatory cascade response, immune cell function, and endothelial damage repair, which provides a theoretical basis at the molecular level for elucidating its neuroprotective effects. TNF, IL-6, and IL-1β, key pro-inflammatory cytokines in SAE, drive neuronal injury and cerebral edema via NF-κB activation (Guo et al., 2024). IL-6 and IL-1β exacerbate BBB permeability, facilitating leukocyte infiltration and brain injury (Yang et al., 2013). Magnesium sulfate may attenuate neuroinflammation by suppressing TNF, IL-6, and IL-1β expression. Prior studies confirm magnesium sulfate inhibits NF-κB activation, reducing cytokine release (Gao et al., 2013; Ashique et al., 2023). CXCL8 recruits neutrophils, amplifying neuroinflammation (Hussain et al., 2025), while MMP9 degrades extracellular matrix, disrupting BBB integrity (Ulbrich et al., 2021). Magnesium sulfate downregulates CXCL8 and MMP9, mitigating leukocyte infiltration and tissue damage, as evidenced by its ability to reduce MMP9 expression and restore BBB function in preclinical models (Shadman et al., 2019). ICAM1 and VCAM1, endothelial adhesion molecules, promote leukocyte-endothelial adhesion in SAE (Kong et al., 2018; Otgongerel et al., 2023). Magnesium sulfate likely inhibits their expression, attenuating neuroinflammation.

TGFB1 modulates immune balance and tissue repair, with dysregulation contributing to SAE pathology (Rodari et al., 2022; Deng et al., 2024). Magnesium sulfate may restore TGFB1-mediated immune homeostasis. ALB, a critical plasma protein, correlates with sepsis severity and inflammation (van de Wouw and Joles, 2022; Cao et al., 2023). Magnesium sulfate’s modulation of ALB metabolism may alleviate sepsis-induced metabolic disturbances. These findings suggest that magnesium sulfate administration should be individualized based on risk stratification. Patients with hypomagnesemia, vasopressor requirements, or biomarkers indicative of neuroinflammation (e.g., elevated IL-6, MMP9) may derive greater benefit, whereas those with hypermagnesemia or advanced renal dysfunction require cautious dosing. Prospective trials should validate these subgroups to optimize clinical protocols. As for its clinical considerations and safety, the use of magnesium sulfate requires vigilance in ICU settings. Rapid infusion may induce hypotension or arrhythmias, particularly in hemodynamically unstable patients (Da et al., 2020). Hypermagnesemia (serum Mg²^+^ >2.5 mmol/L) must be avoided in patients with renal impairment, necessitating frequent monitoring and dose adjustment during CRRT. Contraindications include severe bradycardia, neuromuscular disorders, and concurrent use of calcium channel blockers due to their potential for additive cardiovascular effects.

By integrating MIMIC-IV data with network pharmacology, this study elucidates magnesium sulfate’s potential mechanisms in SAE, emphasizing neuroinflammation suppression and BBB stabilization. These findings provide a theoretical foundation for optimizing SAE treatment. While this study provides valuable insights into the potential mechanisms and clinical benefits of magnesium sulfate in SAE, several limitations should be considered. The retrospective nature of the analysis introduces potential biases, including unmeasured confounders and the inability to establish definitive causality. Our findings are based on the MIMIC-IV database, which, despite its comprehensiveness, may not fully represent the diversity of global SAE patient populations. This could affect the generalizability of our results to other settings or patient groups. Additionally, network pharmacology predictions require experimental validation, and the complexity of *in vivo* environments may not be fully replicated. The potential drug - drug interactions in clinical settings were also not addressed in this study. The current lack of human SAE brain tissue transcriptomic data in publicly available databases that meet stringent diagnostic criteria forced us to rely primarily on mouse models for mechanistic exploration. Future research should include prospective, multi - center trials to validate these findings and further investigate the safety and efficacy of magnesium sulfate in diverse clinical settings.

## 5 Conclusion

In the ICU setting, magnesium sulfate use was significantly associated with reduced ACM in patients with SAE. Magnesium sulfate exerts its therapeutic effects by targeting core mediators, including TNF, IL6, IL1B, CXCL8, TGFB1, ICAM1, VCAM1, MMP9, ALB, and NFKB1. These multi-target mechanisms involve suppression of pro-inflammatory cytokine cascades, attenuation of extracellular matrix degradation, and modulation of immune balance. These findings provide a mechanistic basis for magnesium sulfate's neuroprotective effects in sepsis-associated encephalopathy (SAE) and offer strategic directions to refine its clinical application protocols.

## Data Availability

Publicly available datasets were analyzed in this study. This data can be found here: The data that support the findings of this study are openly available in MIMIC-IV database at https://mimic.mit.edu. Data for this study were collected by the author, B.C., who holds a certification from the Collaborative Institutional Training Initiative (CITI) with the identification number 65165972.
