# Supplementary material for: A multi-omics study of magnesium sulfate to improve prognosis in sepsis-related encephalopathy: integrating clinical data-driven network pharmacology
Source: Front Cell Infect Microbiol. 2025 Jun 9;15:1607586. doi: 10.3389/fcimb.2025.1607586 (PMC12183159; doi:10.3389/fcimb.2025.1607586)
Supplement: Supplementary file 1 [file Supplementaryfile1.docx]

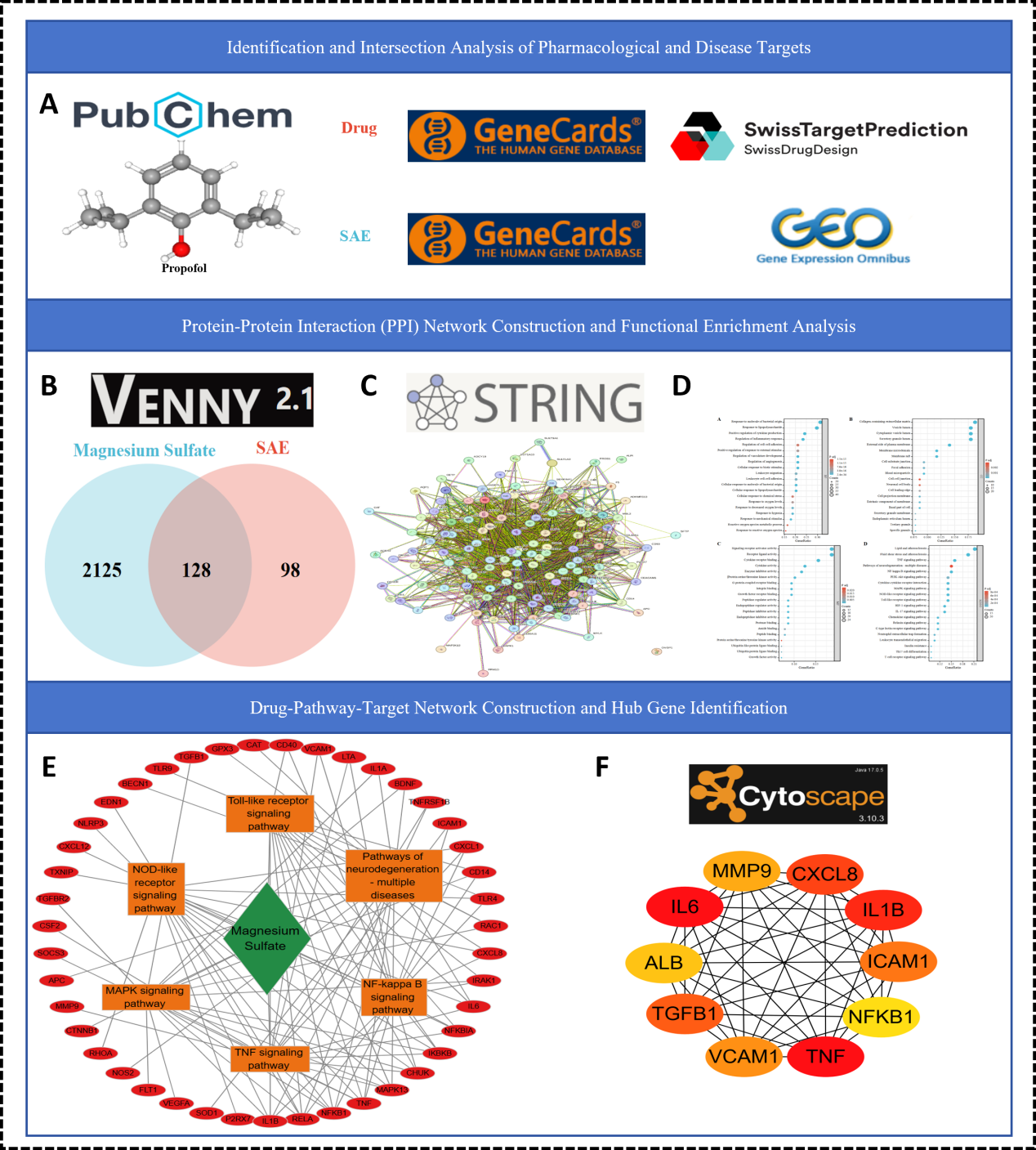


Supplementary Figure 1. Workflow of Network Pharmacology


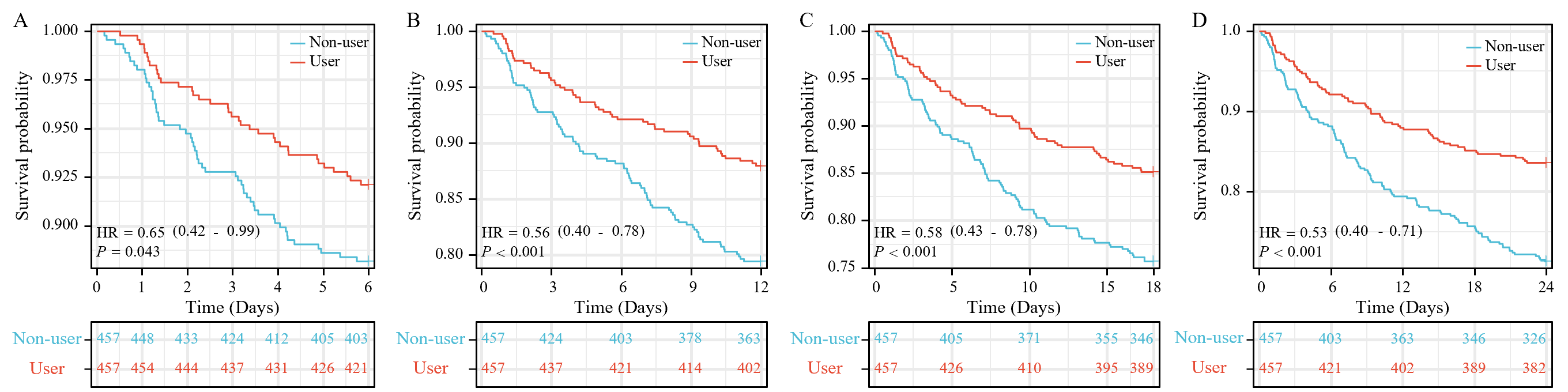


Supplementary Figure 2. KM curve analysis of 6 -, 12 -, 18 -, and 24 -day survival rates in SAE patients who did and did not receive magnesium sulfate. (Survival rates were calculated by the Kaplan-Meier method, based on time of death or study time node, and were not adjusted for covariates.)


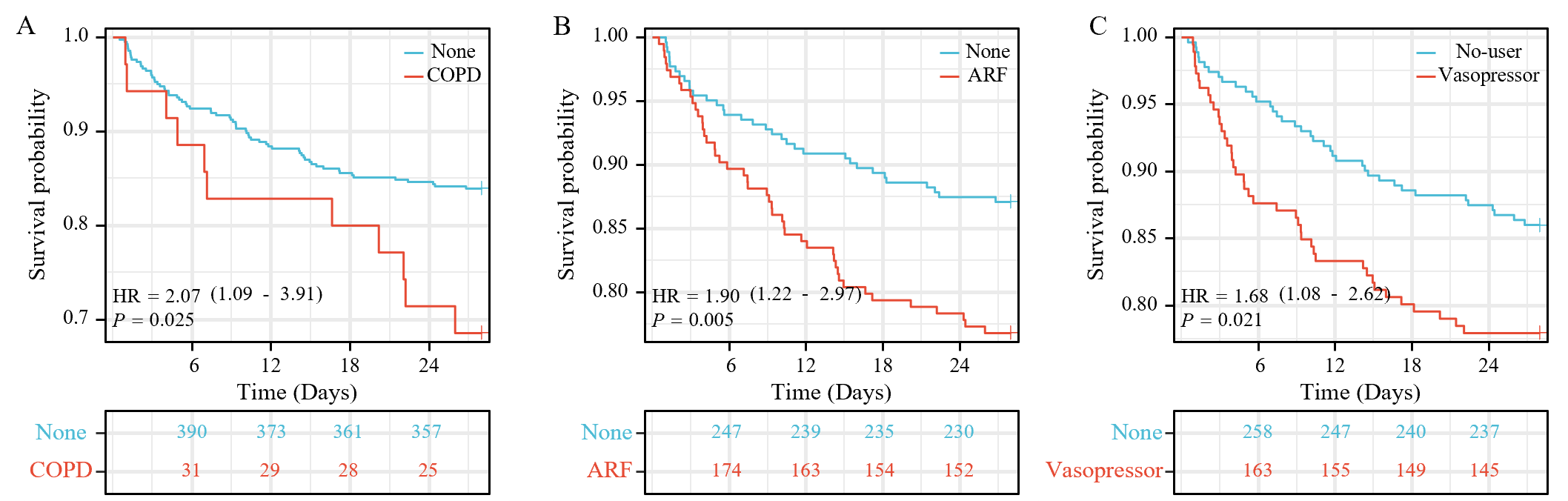


Supplementary Figure 3. KM curve analysis of magnesium sulfate use and 28-day survival in selected SAE patients
